# Evolution of polyamine resistance in *Staphylococcus aureus* through modulation of potassium transport

**DOI:** 10.1128/msphere.00613-24

**Published:** 2025-08-18

**Authors:** Killian Campbell, Caitlin H. Kowalski, Kristin M. Kohler, Mara R. Kebret, Matthew F. Barber

**Affiliations:** 1 Institute of Ecology and Evolution, University of Oregon 209847 , Eugene, Oregon, USA; 2 Department of Biology, University of Oregon 209846, Eugene, Oregon, USA; University of Nebraska Medical Center College of Medicine, Omaha, Nebraska, USA

**Keywords:** *Staphylococcus aureus*, polyamines, experimental evolution, potassium, antibiotics

## Abstract

Microbes must adapt to diverse biotic and abiotic factors encountered in host environments. Polyamines are an abundant class of aliphatic molecules that play essential roles in fundamental cellular processes across the tree of life. Surprisingly, the bacterial pathogen *Staphylococcus aureus* is highly sensitive to polyamines encountered during infection, and acquisition of a polyamine resistance locus has been implicated in the spread of the prominent USA300 methicillin-resistant *S. aureus* lineage. At present, alternative pathways of polyamine resistance in staphylococci are largely unknown. Here, we applied experimental evolution to identify novel mechanisms and consequences of *S. aureus* adaptation when exposed to increasing concentrations of the polyamine spermine. Evolved populations of *S. aureus* exhibited striking evidence of parallel adaptation, accumulating independent mutations in the potassium transporter genes *ktrA* and *ktrD*. Mutations in either *ktrA* or *ktrD* are sufficient to confer polyamine resistance and function in an additive manner. Moreover, we find that *ktr* mutations provide protection against multiple classes of unrelated cationic antibiotics, suggesting a common mechanism of resistance. Consistent with this hypothesis, ktr mutants exhibit alterations in cell surface charge indicative of reduced affinity and uptake of cationic molecules. Finally, we observe that laboratory-evolved *ktr* mutations are also present in diverse natural *S. aureus* isolates, suggesting these mutations contribute to antimicrobial resistance during human infections. Collectively, this study identifies a new role for the *S. aureus* potassium transport system in resistance to both host-derived and clinically used antimicrobials.

**IMPORTANCE:**

*Staphylococcus aureus* is a leading cause of infectious disease-related deaths globally. Understanding factors that govern adaptation and survival of *S. aureus* and other pathogens in the host environment is critical for improving infection outcomes. It has been known for several years that *S. aureus* is highly sensitive to polyamines, a broadly produced class of molecules that play important cellular functions across bacteria and eukaryotes. How *S. aureus* is capable of adapting to polyamine toxicity remains largely mysterious. Using experimental evolution, our study reveals that changes in potassium transport are sufficient to confer high-level polyamine resistance in *S. aureus* while simultaneously increasing resistance to unrelated classes of clinically used antibiotics. Our results identify new roles for bacterial potassium transport in polyamine resistance as well as highlighting the utility of experimental evolution for identifying new genetic determinants of pathogen adaptation.

## INTRODUCTION

*Staphylococcus aureus* is a Gram-positive bacterium that colonizes approximately 30% of the human population as well as human-associated animals (1, 2). *S. aureus* is most frequently isolated from the anterior nares, where it resides as a commensal member of the human microbiota (3, 4). However, *S. aureus* is also capable of colonizing the skin and is a leading cause of soft skin and tissue infections (SSTIs) as well as other invasive conditions including bloodstream infections, pneumonia, osteomyelitis, and endocarditis (4–8). *S. aureus* is a frequent agent of antibiotic-resistant infections, with methicillin-resistant *S. aureus* (MRSA) causing between 10,000 and 20,000 deaths in the United States annually (8, 9). Recent global estimates indicate that *S. aureus* is a leading cause of both total bacterial infections and antibiotic-resistant infections in humans across geographic and socioeconomic boundaries (10, 11).

Previous studies have identified a number of biotic and abiotic factors that limit *S. aureus* colonization in distinct host niches (12–15). In particular, polyamines are an abundant class of aliphatic cationic molecules known to restrict *S. aureus* growth during infection (16, 17). Polyamines are characterized by the presence of at least two primary amine groups and include putrescine, agmatine, spermidine, and spermine (18, 19). Polyamines are byproducts of arginine metabolism and are synthesized by different series of enzymatic steps, depending on the organism (20). Polyamine metabolism and functions are well-studied in eukaryotes, where they play pleiotropic roles in cellular physiology (21–23). Due to their net positive charge, polyamines are capable of binding to ribosomes and DNA during translation and DNA replication, respectively (18, 24). In mammals, the enzymes that produce and break down polyamines are controlled at multiple transcriptional levels, resulting in a tightly regulated pool of polyamines within a cell at any given time (19, 25).

Given the numerous roles that polyamines play in essential cellular functions, polyamine synthesis was once believed to be conserved across the entire tree of life (26). This dogma was overturned by work demonstrating that multiple bacterial genera have lost the ability to synthesize polyamines *de novo* (21). Even among bacteria that do not synthesize polyamines, supplementing media with exogenous polyamines often results in modest growth improvement (27, 28). In marked contrast to other bacteria, polyamines are highly bactericidal to most *Staphylococcus* species with particularly potent effects on *S. aureus* (16). A key exception are *S. aureus* strains within the USA300 lineage, which exhibit enhanced polyamine resistance (28, 29). USA300 defines a lineage of community-acquired MRSA (CA-MRSA) that emerged in the early 2000s that has since spread to epidemic levels (29). The discovery of the arginine-catabolic mobile element (ACME) unique to USA300 provided a key genetic determinant that has likely contributed to the rise of this lineage (28–30). ACME is an approximately 31 kb genomic island that contains at least 33 putative genes (28, 29). Evidence suggests that ACME was assembled in *Staphylococcus epidermidis* and then horizontally transferred to the most recent common ancestor of USA300 (28). Notably, ACME contains a spermine acetyltransferase gene, *speG*, which is sufficient to confer resistance to polyamines (16, 28). While acetyltransferase activity of *speG* is required for polyamine resistance (16), how acetylation of polyamines reduces their toxicity to *S. aureus* remains unresolved.

The ACME locus contains several genes related to arginine metabolism, which produce ammonia allowing these strains to efficiently colonize and infect the acidic environment of the skin (16, 28, 29, 31). At present, the basis of polyamine toxicity and mechanisms by which non-USA300 *S. aureus* may adapt to these molecules is largely unknown. While ACME and *speG* in particular provide an example of how polyamine resistance may evolve, alternative genetic or molecular mechanisms of resistance have not been well-characterized. To address this question, we experimentally evolved populations of *S. aureus* under increasing concentrations of spermine and surveyed the consequences of resistance evolution across three different strain backgrounds. Our findings identify new genes and mechanisms contributing to polyamine resistance in this major bacterial pathogen.

## RESULTS

### Experimental evolution of spermine resistance in *S. aureus*

**Fig 1:**
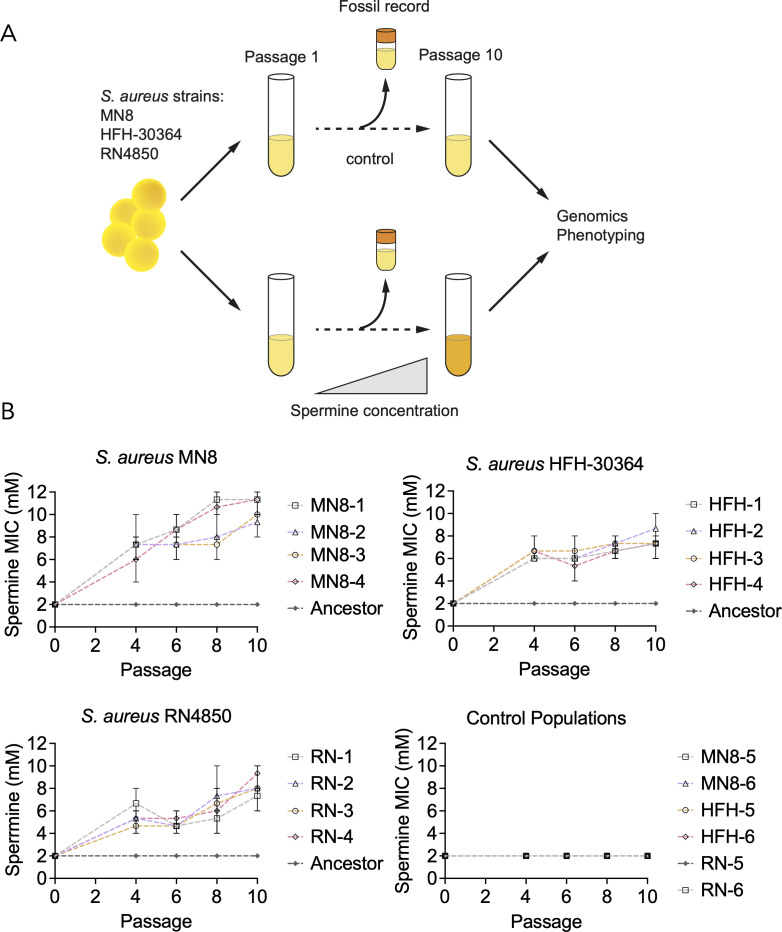
Experimental evolution of spermine resistance in *Staphylococcus aureus*. (**A**) Experimental evolution overview. Populations of each strain of *S. aureus* (MN8, HFH-30364, and RN4850) were revived from glycerol stocks onto tryptic soy agar (TSA). For each strain background, four single colonies were picked to establish replicate populations to undergo exposure to spermine, and two colonies were picked to establish replicate control lines to be passaged in TSB alone. Populations were passaged at a fixed dilution daily, and spermine-exposed lines were continuously exposed to spermine. Glycerol stocks containing aliquots of the evolving populations were saved periodically as a “fossil record.” At the end of the experiment, glycerol stocks were revived to perform phenotypic analyses and to isolate DNA for sequencing. (**B**) Spermine minimal inhibitory concentration (MIC) was calculated over the course of the evolution experiment. Glycerol stocks from throughout the experiment were revived on TSA plates, then single colonies were picked and spermine MIC was measured. Shapes and error bars represent average and range of MIC values measured. Each point represents the average of a separate experiment conducted in triplicate.

To identify mechanisms of polyamine resistance in *S. aureus*, we performed a serial-passaging experiment in which populations of *S. aureus* were continuously exposed to increasing concentrations of spermine (Fig. 1A; Table S1). Initially, single colonies of *S. aureus* strains MN8, HFH-30364, and RN4850 were revived from glycerol stocks on tryptic soy agar (TSA) plates. Both *S. aureus* MN8 and RN4850 are methicillin-sensitive strains, while HFH-30364 is a MRSA isolate from the USA400 lineage (Table 1). Replicate populations of each strain were then inoculated in tryptic soy broth media (TSB) to perform the passaging experiment. Spermine was selected as a representative polyamine as it has a well-documented bactericidal effect on *S. aureus* (16) and is abundant during the post-inflammatory phase of wound healing in murine models (17). Each day, a 1/100 aliquot of each replicate culture was transferred to fresh TSB with a pre-determined concentration of spermine (or no spermine for the control populations). Populations were initially exposed to sub-inhibitory conditions of spermine (2 mM) and passaged daily for 10 days, until reaching a final concentration of 7 mM spermine. Genomic DNA was collected to perform whole-population sequencing of MN8 samples taken throughout the evolution experiment. For RN4850 and HFH-30364, we conducted whole-genome, population-level sequencing upon completion of the passaging experiment (Fig. 1A).

**TABLE 1:** Mutations observed during experimental evolution^*a*^

Population	*Ktr* potassium transport complex		ATP synthase machinery			*pgl*
	*ktrA*	*ktrD*	*atpG*	*atpA*	*atpH*	
MN8-1	E76G	Q450K, S135L	–	–	–	–
MN8-2	–	–	–	–	–	–
MN8-3	E76G	M391I, T277N	–		–	–
MN8-4	A178V	G94C	–	–	–	–
HFH-1	–	–	–	P95L	–	G4E, H306Y
HFH-2	E151V	–	T242P	–	–	G108V
HFH-3	–	–	–	–	–	G287V, G323D
HFH-4	–	–	–	–	–	G4E, G120S, R318S
RN-1	–	–	–	–	–	A245V, T10P
RN-2	–	–	∆69 bp	–	v	P234L, S41F, G261E, H138Y, F268V
RN-3	–	–	L261*	–	–	A245V
RN-4	–	–	–	–	Q170*	–

^*a*^ A subset of mutations that arose in each lineage of each strain background during the evolution experiment. Mutations were detected using breseq from genome sequencing of DNA isolated at passage 10 of the evolution experiment. “*” denotes premature stop codon mutations. “–” indicates no mutation.

We observed that all populations in each of the three strain backgrounds evolved increased spermine resistance within 10 passages of laboratory evolution. Specifically, both *S. aureus* MN8 and HFH-30364 populations evolved levels of resistance at least fourfold greater than the minimal inhibitory concentration (MIC) of the ancestral strain (Fig. 1B). *S. aureus* RN4850 exhibited a more variable resistance profile, with most replicate populations evolving an MIC nearly four times the ancestral levels (Fig. 1B). Individual colonies tested from each population displayed slight differences in spermine resistance, likely reflecting heterogeneity in mutations carried by individual clones. *S. aureus* MN8 displayed the largest fold change in spermine MIC, with individual colonies measuring between 5× and 6× the ancestral MIC levels (Fig. 1B). We noted that the dynamics of resistance evolution as measured by spermine MIC varied between strain backgrounds. *S. aureus* MN8 gradually evolved increased resistance throughout the passaging experiment, while HFH-30364 experienced a single, large increase in resistance change between passages 0–4, and RN4850 experienced two large increases in resistance from passages 0–4 and passages 8–10 (Fig. 1B). Together, these results reveal that *S. aureus* can readily evolve resistance to spermine under laboratory conditions, while the dynamics of resistance evolution differ between strain backgrounds (Fig. 1B). Given that *S. aureus* MN8 populations evolved the highest magnitude change of resistance relative to the ancestor, we chose to focus on characterizing the genetic basis of spermine resistance in this strain background for future experiments.

### Genetic determinants of spermine resistance

We next sought to determine the genetic basis of polyamine resistance in our evolved *S. aureus* populations. Whole-population DNA samples were extracted from each replicate population throughout the experiment (MN8) and at the end of the passaging experiment (HFH-30364, RN4850). Additionally, we extracted DNA from ancestral clones to generate a reference sequence for variant calling. *S. aureus* MN8 populations exhibited striking evidence of convergent evolution, with three of the four spermine-exposed populations harboring mutations in different components of the *ktr* potassium transport complex (Table 1). The Ktr system has been characterized in *Bacillus subtilis* as a constitutively expressed moderate-affinity potassium transporting system and has only recently been described in *S. aureus* (32, 33). This complex is composed of three proteins: KtrA, which forms a regulatory octameric ring subunit in the cytoplasm, and two redundant, ion-conducting proteins: KtrB and KtrD. Each is dimeric transmembrane channels that facilitate potassium import. KtrB or KtrD, in combination with KtrA, is required to form a functional complex. Previous work has implicated the Ktr complex in both osmotic and alkaline stress tolerance (32–34). The Ktr complex also contributes to survival against a variety of antibacterial agents under low potassium conditions, as Δ*ktrA* deletion mutants possess increased sensitivity to aminoglycoside antibiotics (32, 33). Despite this, no study to date has directly linked Ktr complex function and polyamine resistance.

**Fig 2:**
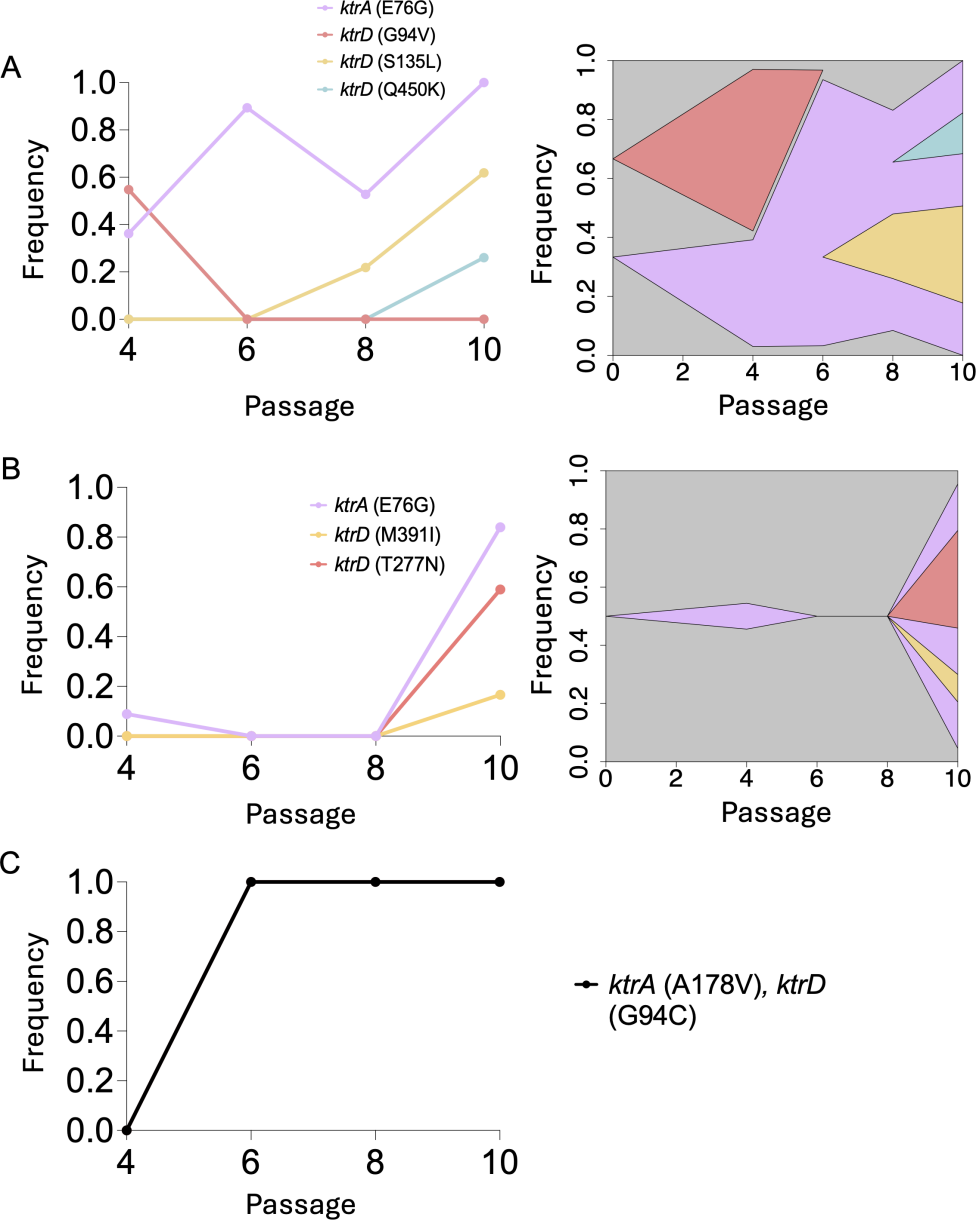
Allele frequency dynamics and linkage of *ktr* mutations over the course of experimental evolution. Allele frequencies and linkage were determined for *ktrA* and *ktrD* mutations in the MN8-1 (**A**), MN8-3 (**B**), and MN8-4 (**C**) populations. For all allele frequency line plots, breseq was run in polymorphism mode to determine the frequency of *ktrA* and *ktrD* single nucleotide polymorphisms (SNPs) detected in the population throughout the evolution experiment. To determine the linkage of *ktr* alleles present in each population, a combination of PCR and Sanger sequencing was used. For both MN8-1 and MN8-3, 16 different colonies were picked, and colony PCR was conducted on both the *ktrA* and *ktrD* locus for each colony. PCR products were sent for Sanger sequencing, and sequences were aligned to detect which SNPs were present in each clone. Sanger sequencing revealed that clones in both MN8-1 and MN8-3 could contain a single SNP in *ktrD* on the background of a single SNP in *ktrA*.

MN8-derived populations exhibited the highest levels of polyamine resistance of the three strain backgrounds tested and were the only populations carrying high-frequency mutations in *ktr* genes (Table 1). We were therefore motivated to further investigate the evolutionary dynamics of spermine resistance in the MN8 populations. We leveraged whole-genome, whole-population sequencing to track the frequencies of *ktr* mutations over time. In each of the three replicate populations containing *ktr* mutations (MN8-1, MN8-3, MN8-4), nonsynonymous mutations occurred in both *ktrA* and *ktrD* at high frequencies by the final passage (Fig. 2). Specifically, a single nonsynonymous mutation in *ktrA* had fixed by passage 10 in replicate populations MN8-1 and MN8-4 and had nearly fixed in MN8-3 (84% allele frequency) (Fig. 2A and C).

Notably, only a *single ktrA* allele was maintained in any of the populations by the final passage, whereas multiple *ktrD* alleles co-occurred in both MN8-1 and MN8-3 (Fig. 2A and B). The allele frequency dynamics were also different throughout each of the replicate populations. Single mutations in *ktrA* and *ktrD* arose early and fixed in MN8-4 by passage 6 (Fig. 2C). However, the dynamics for MN8-1 and MN8-3 was more varied. In MN8-1, a single mutation in *ktrA* largely persisted until passage 8, when multiple *ktrD* mutations were detected in the population at low frequencies (Fig. 2A and B). MN8-3 displayed similar characteristics to MN8-4, in which all *ktr* mutations arose late in the experiment between passages 8 and 10 (Fig. 2B and C). We next picked multiple clones from the terminal passage in both MN8-1 and MN8-3 to determine the linkage of these *ktr* mutations. While multiple mutations were present in the *ktrD* gene in MN8-1, they existed in different lineages and always in combination with the *ktrA* mutation (Fig. 2A). This was also true in MN8-3, where a single mutation in *ktrD* was found in combination with a single mutation in *ktrA* (Fig. 2B). Despite multiple *ktrD* mutations existing at the population level, we did not detect any lineages within MN8-3 carrying multiple *ktrD* mutations (Fig. 2B). Our findings suggest that resistance to spermine was largely conferred through a combination of single mutations in both *ktrA* and *ktrD*.

### Single amino acid changes in *ktr* genes confer additive polyamine resistance

**Fig 3:**
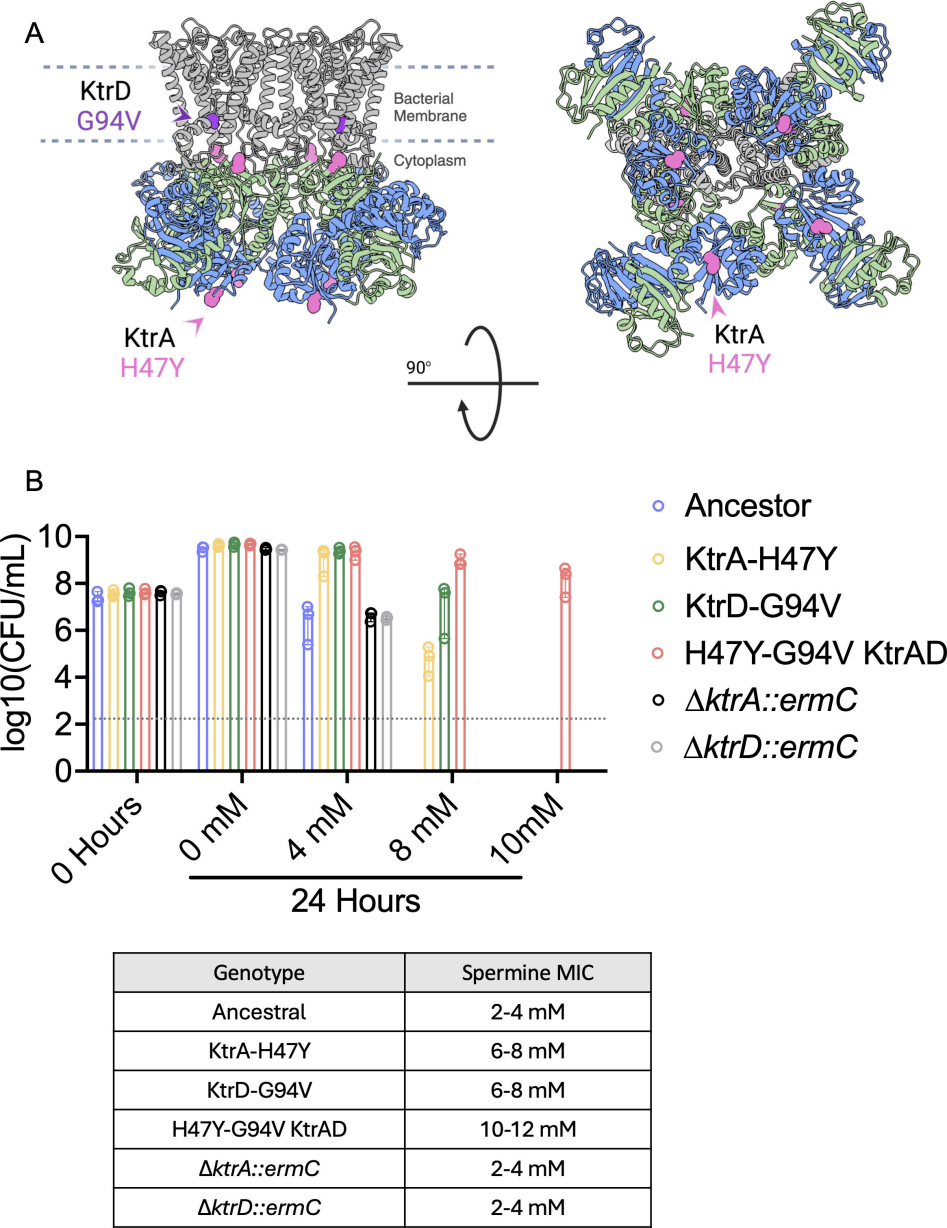
Evolved mutations in the KtrAD complex confer additive spermine resistance in *S. aureus*. (**A**) Predicted structure of the KtrAD complex generated using ChimeraX. *S. aureus ktrA* and *ktrD* gene sequences were used to model the KtrAD complex based on the published KtrAD crystal structure from *Bacillus subtilis* (PDB ID: 4J7C). Mutations introduced into the MN8 strain background are highlighted in pink (KtrA) and purple (KtrD). (**B**) Colony-forming units (CFUs) recovered from MN8 *S. aureus* strains incubated in different concentrations of spermine for 24 h (top), with corresponding spermine MIC values listed in the table (bottom). A gray dotted line represents the limit of detection.

To determine whether single mutations in *ktr* genes are sufficient to confer spermine resistance in *S. aureus*, we generated targeted mutations in both *ktrA* and *ktrD* by homologous recombination in strain MN8. Specifically, the histidine at position 47 of KtrA was substituted for a tyrosine (H47Y), and the glycine at position 94 of KtrD was substituted for a valine (G94V) (Fig. 3A). These mutations were recovered during a pilot evolution experiment performed as described in Fig. 1 (Table S2) and are in proximity to mutations recovered in the evolution experiments described above. We then measured spermine MIC of each single mutant, as well as the *ktrA/ktrD* double mutant. We found that each single mutation conferred partial resistance to spermine, with an MIC of 6–8 mM for both the KtrA H47Y and KtrD G94V alleles (Fig. 3B). However, the *ktrA/ktrD* double mutant conferred a similar level of resistance to spermine that we observed at the final passage of our evolution experiment (7 mM), making this combination of mutations sufficient to provide the level of resistance observed in these populations (Fig. 3B). Together, these results demonstrate that evolved mutations in *ktrA* and *ktrD* are sufficient to confer polyamine resistance in *S. aureus* in an additive manner. We also confirmed the ability of these strains to survive spermine concentrations by enumerating colony-forming units per milliliter of culture (CFU/mL) (Fig. 3B). We observed a ~ 100-fold reduction in recovered CFUs for the ancestral strain when incubated for 24 h in 4 mM spermine compared with the no spermine control. In contrast, there was no reduction in CFUs observed for either of the single mutants in 4 mM versus 0 mM spermine (Fig. 3B). We similarly did not observe a significant reduction in CFU/mL for the *ktrA/ktrD* double mutant when incubated with 4 mM or 8 mM spermine, reflecting the ability of single amino acid changes to confer additive spermine resistance (Fig. 3B). Notably, deletion of either *ktrA* or *ktrD* did not alter spermine susceptibility relative to the ancestral MN8 strain (Fig. 3B), indicating that the evolved nonsynonymous mutations do not function as null alleles. Together, these experiments demonstrate that evolved *ktrA* and *ktrD* alleles are sufficient to confer polyamine resistance in *S. aureus*.

### Evolved *ktr* mutations alter *S. aureus* antibiotic susceptibility

**Fig 4:**
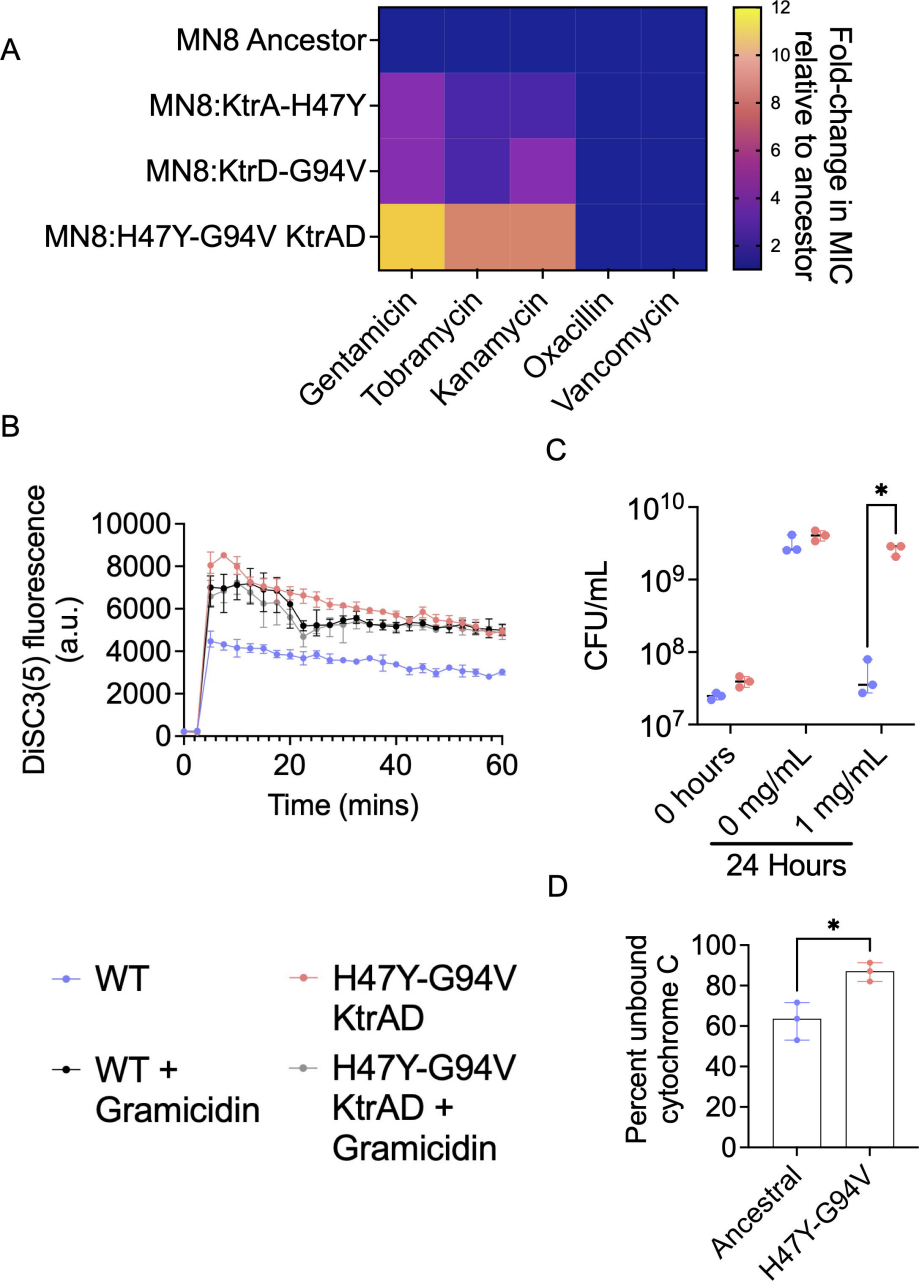
KtrAD complex mutations alter antibiotic resistance, membrane potential, and cell-surface charge. (**A**) Heatmap depicting fold change in MIC for indicated antibiotics in *S. aureus* mutant strains relative to the ancestor. Values represent the average of three experiments each conducted in triplicate. (**B**) 3,3′-Dipropylthiadicarbocyanine Iodide (DiSC3(5)) fluorescence of different engineered strains. Fluorescence readings are normalized to OD600 measurements for each respective strain. Points on the graph each represent the average of a different experiment conducted in triplicate. (**C**) CFUs of the ancestral MN8 strain or double *ktrAD* mutant measured after 24 h of incubation with polymyxin B. Points represent average of a different experiment conducted in triplicate. Asterisks represent significant difference (*P* < 0.05) in means between two groups compared as detected by an unpaired *t*-test. (**D**) Percent unbound cytochrome C was measured via absorbance at 410 nm in a spectrophotometric plate reader. Percent unbound cytochrome C was calculated relative to the absorbance of cytochrome C alone in buffer without cells. Asterisks represent significant difference (*P* < 0.001) as determined by an unpaired *t*-test.

Previous reports indicate that the *ktr* potassium transport system plays an important role in antimicrobial resistance and alkaline stress tolerance in *S. aureus* (32, 33). We therefore sought to determine if our evolved *ktr* mutations also altered the ability of *S. aureus* to tolerate other stressors beyond spermine. We measured the change in MIC for a panel of antibiotics in strain MN8 as well as the engineered *ktrA* (H47Y) and *ktrD* (G94V) mutants (Fig. 4A). Previous findings revealed that *ktr* null mutants sensitize *S. aureus* to aminoglycoside antibiotics (32). In contrast to null mutants, the evolved *ktrA/ktrD* double mutant exhibits an approximately 12-fold increase in aminoglycoside MIC compared with the ancestor (Fig. 4A). Aminoglycoside antibiotics rely upon an intact proton motive force to cross the *S. aureus* cell membrane, where they inhibit protein translation (35, 36). We therefore hypothesized that the *ktr* mutants identified through experimental evolution function by altering membrane potential and preventing aminoglycoside internalization. Potassium transport, and *ktr-*mediated potassium transport in particular, is known to regulate membrane potential (32, 37, 38). To address this hypothesis, we used the voltage-sensitive, membrane-permeable dye, DiSC_3_(5) that readily enters *S. aureus* cells in a polarized state. Depolarization of the cell membrane prevents efficient uptake of the dye. We detected a modestly depolarized membrane potential in the *ktr* double mutants compared to the ancestral strain (Fig. 4B). Despite reproducible differences in membrane potential, we were not convinced that this was the only factor contributing to antimicrobial resistance in the *ktr* mutants due to effect size. Prior to internalization into the bacterial cell, aminoglycoside uptake is governed by electrostatic attraction between the positively charged antibiotic and negatively charged bacterial cell wall (39). Given the polycationic nature of both spermine and aminoglycosides at physiological pH and the observed resistance of the *ktr* mutants to these otherwise unrelated molecules, we hypothesized that an alteration in cell-surface charge, coupled with altered membrane depolarization, could explain the observed change in resistance. To test this hypothesis, we measured survival of the *ktr* double mutant in polymyxin B, an unrelated polycationic antibiotic that has a +5 charge at physiological pH (40). Consistent with our hypothesis, the *ktr* double mutant survived significantly better than the ancestor in the presence of 1 mg/mL polymyxin B (Fig. 4C). To assess changes in bacterial cell surface charge, we measured cytochrome C binding in the ancestral MN8 and *ktr* double-mutant strains (Fig. 4D). Cytochrome C binding provides a convenient proxy to infer bacterial cell-surface charge as it is highly cationic and binds effectively to the negatively charged bacterial cell wall (41). We detected a significant increase in the percent of unbound cytochrome C in the *ktr* mutant when compared with the ancestral strain, consistent with an increase in cell surface charge conferred by *ktr* mutations (Fig. 4D). Taken together, our findings suggest a novel mechanism by which modulation of potassium transport complex function confers resistance to diverse cationic antimicrobial compounds through changes in electrostatic properties of the bacterial cell surface.

### Exogenous potassium is insufficient to confer spermine resistance in *S. aureus*

**Fig 5:**
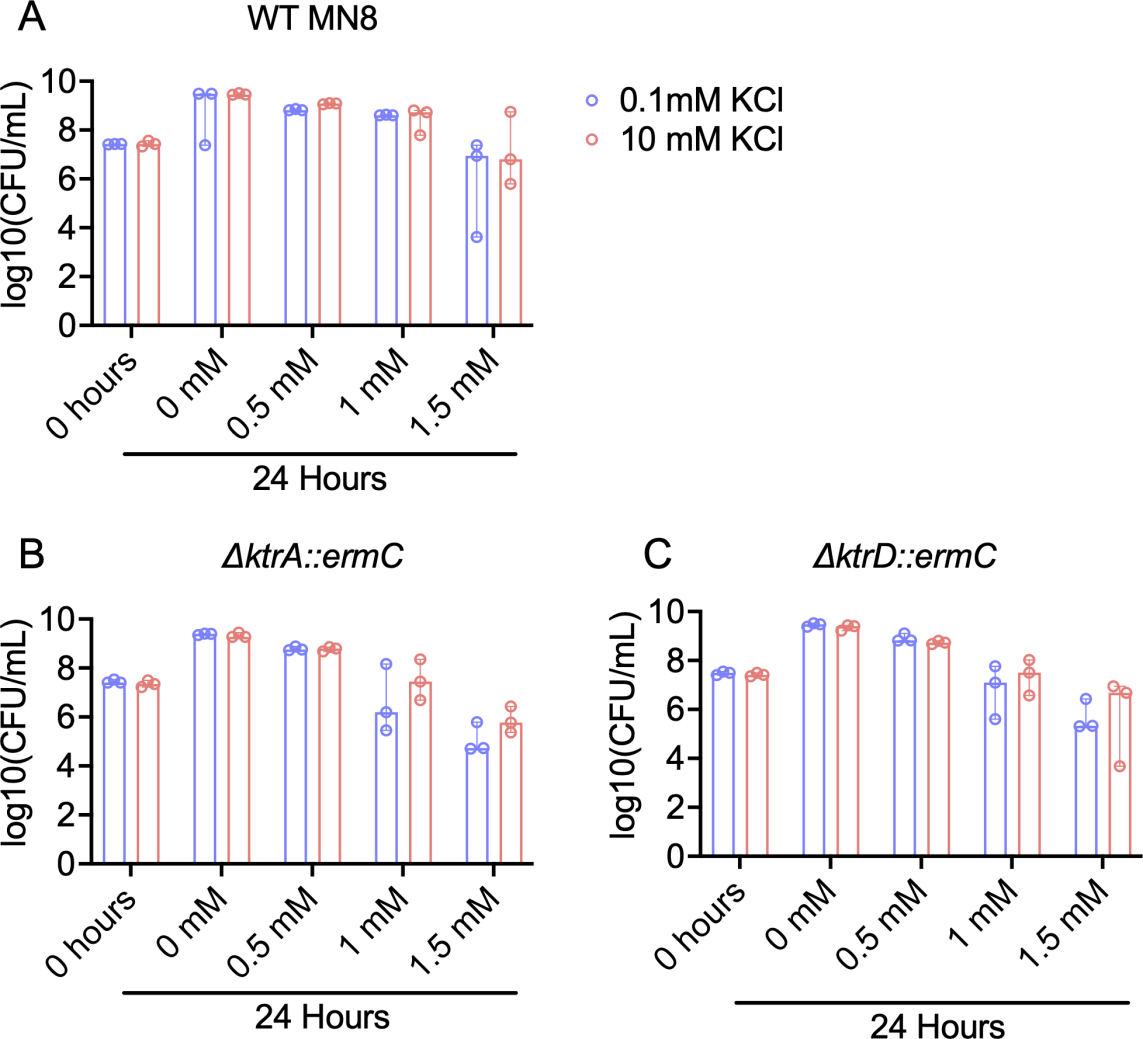
Exogenous KCl supplemented in chemically defined media does not reduce the toxic effects of spermine in *S. aureus* enumerations of colony-forming units after 24 h of incubations in different amounts of spermine in media with high and low amounts of KCl for (**A**) WT *S. aureus* MN8, (**B**) ∆*ktrA::ermC,* or (**C**) ∆*ktrD::ermC* strains.

Given that *ktr* mutants identified in this study exhibit opposite drug resistance phenotypes compared with published *ktr* deletion mutants (32), we hypothesized that our evolved mutants do not result in a complete loss of function. We therefore considered whether changes in exogenous potassium might reveal an effect on spermine toxicity. To test this, we generated a chemically defined medium lacking excess potassium following previously published formulations (33). This allowed us to supplement the growth medium with defined amounts of potassium in the form of KCl. We supplemented the media with physiologically relevant amounts of potassium by adding 0.1 and 10 mM KCl. Exogenous potassium levels had no measurable difference on the amount of CFUs recovered across different concentrations of spermine (Fig. 5). Overall, spermine appears to be slightly more toxic to the cells in this environment; however, this is to be expected for a minimal media in comparison to TSB. This experiment indicates that changes in physiologically relevant potassium levels are not sufficient to alter spermine resistance. We did observe that extremely high levels of potassium (250 mM) appeared to increase resistance to polyamines as well as aminoglycosides (Fig. S5). However, at these levels, it is unclear whether potassium is acting functioning direction through bacterial cells, or rather by interfering with the ability of antimicrobials to interact with the bacterial cell surface. Further work will be necessary in order to more precisely determine the mechanistic basis of evolved mutations on Ktr complex function.

## DISCUSSION

Our study identifies a previously unknown role for the Ktr potassium transport complex in *S. aureus* resistance to diverse cationic antimicrobials. A recent study also recovered mutations in *ktr* genes after passaging *S. aureus* in the presence of a synthetic polyamine, although the effects of these mutations were not reported (42). It is notable that phenotypes conferred by single amino acid changes in the Ktr complex recovered during experimental evolution are markedly different from ∆*ktr* strains described previously (32). Evolved *ktrA* and *ktrD* mutations in this study uniformly decreased sensitivity to aminoglycoside antibiotics and spermine, suggesting a potential alteration of function. Furthermore, the ∆*ktrA* and ∆*ktrD* mutants did not change the sensitivity to spermine relative to the ancestral MN8 strain (Fig. 3). Initially, we hypothesized that the Ktr mutations resulted in increased import of potassium into the cell. However, our experiments supplementing media with KCl and growth in different KCl concentrations do not support this model (Fig. 5; Fig. S1 to S3). The results in this work highlight the necessity to study Ktr-mediated potassium transport across a wider array of *S. aureus* strains. Currently, our understanding regarding the function of the Ktr system in *S. aureus* is based on work in USA300 strains only. Notably, in *S. aureus,* the Ktr complex functions as a unique combination of a single channel regulator (*ktrA*) and multiple ion channels (*ktrB/D)* that are both regulated by *ktrA*. The unique channel architecture suggests that Ktr could provide distinct functions across *S. aureus* isolates that differ from those characterized in other bacteria. The same evolved mutations in *ktr* genes also altered membrane potential and cell-surface charge, which may contribute to spermine and aminoglycoside resistance. Links between potassium transport and membrane potential are well-documented, although there is a lack of evidence in the literature regarding the role of potassium transport in regulating bacterial cell surface charge (43). Notably, a previous study also found that potassium transport can alter zeta potential (the voltage field that arises from a cell surface) in a red blood cell model (44). Work is ongoing to clarify the connections between Ktr complex function, bacterial physiology, and resistance to antimicrobial agents. Notably, the relationship between cations, polyamines, aminoglycosides, and Gram-positive bacteria has been noted previously (45). This work demonstrated that aminoglycosides, spermidine, and magnesium cations compete for binding wall-teichoic acids on Gram-positive bacterial cell walls. Future studies could aid in further clarifying how alterations in cell envelope architecture influence susceptibility to polyamines and other cationic antimicrobials.

How independent mutations in the Ktr complex together act to enhance the resistance phenotype remains unknown. It is possible that these mutations increase the permeability or sensitivity of the transporter complex. Mutations were identified in different regions of each subunit and within the complex, suggesting that there is not a single interaction interface that is being modified by these mutations. Further mechanistic studies of Ktr complex mutations and their biochemical effects could aid in resolving these questions, as well as unraveling the broader connections between potassium transport and antimicrobial resistance.

The observation that natural *S. aureus* isolates carry identical mutations in Ktr complex genes as those recovered in our experiments suggests that changes in potassium transport may confer important fitness advantages during human colonization or infection (Table S3). The fact that these mutations are observed repeatedly but do not appear to be fixed in major *S. aureus* lineages suggests that they have arisen *de novo* during human colonization or infection. This would be consistent with other drug resistance mutations that provide a fitness benefit under certain conditions but also carry fitness trade-offs in the absence of drug. Notably, we find that Ktr mutations provide enhanced resistance to diverse cationic antimicrobials, potentially through alterations in bacterial cell surface charge (Fig. 4). In *S. aureus,* changes in cell surface charge can reduce susceptibility to both daptomycin and vancomycin, two antibiotics of choice for treating MRSA infections (46, 47). Additionally, cell surface charge alterations can promote defense against host-encoded antimicrobial peptides, suggesting a potential strategy for *S. aureus* to establish an infection in the host environment (47, 48). Despite this, mutations in the Ktr complex come at a slight fitness cost, primarily as an extended lag phase in rich media (Fig. S3). Further studies to understand the contribution of the Ktr complex function during human infections or drug resistance could aid in addressing these questions.

Our findings illustrate how combining laboratory experimental evolution with natural strain population genomics can provide unique insights on the genetic basis of pathogen adaptation. In this study, mutations in *ktrA* and *ktrD* were detected in the MN8 strain background (Table 1). Conversely, mutations in *pgl,* a gene that encodes for a lactonase and acts in the pentose phosphate pathway, were not recovered in MN8, frequently recovered in both HFH-30364 and RN4850 strains (Table 1; Tables S5 to S16). Notably, mutations in *pgl* are known to affect cell wall-specific phenotypes, such as sensitivity to β-lactam antibiotics and cell wall-permeable compounds (49, 50). In addition, *pgl* mutations increase the cell surface charge of *S. aureus*, similar to our observations in evolved *ktr* mutants (49, 50). This data suggests the potential for convergent evolution of spermine resistance at the mechanistic level, albeit via different genetic mechanisms. Future work could investigate if strain-specific differences in cell-wall composition predispose antimicrobial resistance to evolve via mutations in either *ktr* or *pgl*. Why mutations in *ktrA* and *ktrD*, but not *ktrB*, were recovered during our experiments is unclear. This could reflect unique differences in expression or regulation of these subunits in *S. aureus* or within particular strains. Alternatively, these observations could be due to as-yet undescribed functional differences in *ktrB* and *ktrD* function within the complex. Additional work leveraging targeted mutagenesis could aid in resolving potential unique roles of these Ktr complex subunits.

While this study has identified new bacterial genes and pathways that contribute to polyamine resistance, the mechanism underlying polyamine toxicity in *S. aureus* remains mysterious. Previous efforts to characterize spermine toxicity revealed the gene *menD* as a potential target, revealing the electron carrier menadione as an important factor in mediating toxicity (16, 51). This study found that inactivation of *menD,* the gene that encodes for a menaquinone biosynthetic protein in *S. aureus,* increased resistance to spermine under aerobic conditions. While we did not recover mutations in *menD* during experimental evolution, it is possible that inactivating *menD* similarly alters the membrane potential and cell-surface charge of *S. aureus,* indicating a similar mechanism of resistance (52, 53). Alternatively, it is possible that menadione interacts with polyamines to produce a toxic byproduct that kills *S. aureus* (16). Questions also remain regarding the mechanism of polyamine resistance provided by *speG*. While *speG* homologs are known to acetylate polyamines and acetyltransferase activity is required for *speG*-mediated polyamine resistance, it is unclear specifically how acetylation counteracts polyamine toxicity. Although acetylation partly reduces the positive charge of polyamines, they also exert stronger antimicrobial activity at high pH when their positive charge is reduced (16, 50). Ultimately, the mechanism of bacterial killing by polyamines will continue to be an important area of investigation and could additionally aid in clarifying mechanisms of evolved polyamine resistance. Together, this study reveals a new role for potassium transport in the unique polyamine susceptibility of *S. aureus*, with consequences for the evolution of multidrug resistance in this major human pathogen.

## MATERIALS AND METHODS

### Strains

All strains used in the experimental evolution experiment were acquired from BEI resources. *Ktr* mutants were generated for the purposes of this study following an allelic exchange protocol described below.

### Experimental evolution

Glycerol stocks of each strain were struck out onto tryptic soy agar (BD) plates and incubated overnight at 37°C. For each strain, a total of six single colonies were picked, and each colony was used to inoculate a separate 3 mL culture containing tryptic soy broth (TSB) (BD). These liquid cultures were grown overnight for approximately 18 h, shaking (225 rpm) at 37°C. Each liquid culture was used to initiate a replicate population in the evolution experiment. Four of the cultures were diluted 1/100 into fresh TSB containing 2 mM of spermine (Sigma, 71-44-3), and two of the cultures were diluted 1/100 into plain TSB as a control. After passaging, the populations were returned to the shaking incubator. The populations were passaged daily in the same 1/100 dilution throughout the course of the experiment. Populations initially exposed to spermine on the first passage were continuously exposed to increasing concentrations of spermine for the remainder of the evolution experiment. Control populations were passaged daily in TSB. Populations were sampled and frozen in a glycerol solution (25% final vol/vol) at passages 0, 4, 6, 8, and 10.

### DNA isolation and sequencing

Whole bacterial populations were sequenced that were exposed to spermine during the experiment, as well as the control populations that were never exposed to spermine. All four replicates of *S. aureus* MN8 populations were sequenced at passages 4, 6, 8, and 10. All four replicate populations of *S. aureus* HFH-30364 and *S. aureus* RN4850 that were exposed to spermine were sequenced at passage 10. For all experimentally evolved populations, ancestral clones for each strain that were used to initiate the evolution experiments were sequenced to provide a reference genome for variant calling. We also sequenced the two control populations (never exposed to spermine) for each strain background at passage 10. To isolate DNA, populations were struck out onto TSA plates from glycerol stocks, and mixed-colony samples were taken liberally from all parts of the plate to capture any diversity potentially present in the population. Bacterial samples were resuspended in TE buffer containing 50 µg/mL lysostaphin and incubated for 1 h to facilitate lysis of cells. DNA was then harvested using a Qiagen DNeasy blood and tissue kit (CAT# 69504) according to manufacturer’s instructions. DNA extracts were sent to SeqCenter (seqcenter.com) for Illumina sequencing.

### Data processing and variant calling

Mutations in the evolved populations were identified with breseq v0.35.7 (54) using the default settings and polymorphism mode to calculate the frequencies of variants detected in the reads. Polymorphism mode in breseq calls a variant in the population if it is observed in both strands of at least 5% of the reads. The average read depth for MN8, HFH-30364, and RN4850 populations was 439×, 410×, and 600×, respectively. The average genome coverage for MN8, HFH-30364, and RN4850 was 99.1%, 99.8%, and 99.5% respectively.

### Minimum inhibitory concentration measurements

Minimum inhibitory concentration (MIC) experiments were conducted using a modified broth microdilution method. To measure the MIC of a given compound, a single colony of bacteria was picked and inoculated into 4 mL of TSB to start overnight cultures. The next day, the OD600 of the cultures was measured and diluted to an OD600 of 0.04. Different concentrations of an antimicrobial compound of interest (e.g. spermine) were made by diluting stocks of the chemical in 2× TSB and water, yielding the final concentrations of the compound in 1× TSB. Then, 50 µL of diluted overnight culture was mixed 1:1 with each concentration of the prepared antimicrobial agent. Mixtures of bacterial cultures and antimicrobial compounds were incubated statically in 96-well plates at 37°C for 24 h. MIC values were determined as a range between the highest concentration at which visible growth occurred, and the lowest concentration at which no visible growth occurred. Bacteria were challenged at each concentration in triplicate, and the results reported are the average of three individual experiments.

### Protein modeling and mutation mapping

Published protein structures (PDB ID: 4J7C) and genetic sequences for the *Bacillus subtilis* KtrAB complex were used as guides to model the structure of the *S. aureus* KtrAD protein complex in ChimeraX (version 1.6.1).

### Generation of *S. aureus* mutants

To generate single base-pair (bp) mutations, allelic exchange was used with the pIMAY vector following protocols previously described (55). PCR products containing sequence approximately 1,000 bp upstream and downstream of the mutation of interest were generated. Primers were designed containing homology arms to facilitate assembly into the cloning vector. Genomic DNA extracted from the evolved MN8 isolates was used as template DNA to amplify the mutations of interest. PCR products were assembled into a digested pIMAY vector using NEBuilder HiFi DNA assembly (CAT# E2621S) according to the manufacturer’s instructions. Assembled vectors were chemically transformed into *E. coli* DC10B and plated onto TSA + 25 µg/mL chloramphenicol to select for successful transformants. Colonies were screened with colony PCR to confirm the presence of the insert gene into the pIMAY vector. Validated plasmids were isolated and electroporated into recipient *S. aureus* strains as described previously (56). Transformed *S. aureus* cells were plated onto TSA + 10 µg/mL chloramphenicol at 28°C overnight. Single colonies were picked and re-plated onto TSA + 10 µg/mL chloramphenicol to select for a single recombination event. Single colonies that grew on chloramphenicol were picked, grown up overnight in plain TSB, and then plated onto TSA + 1 µg/mL anhydrotetracycline (atc) to select for loss of the plasmid backbone. Successful secondary recombinants would be permissive to growth on atc. Single colonies that could grow on ATC were picked and sequenced at the locus of interest to confirm that the mutation of interest was introduced successfully.

### Cytochrome C binding assay

Relative cell surface charge was determined by measuring cytochrome C binding as described previously (57). Stationary phase overnight cultures were harvested and adjusted to an OD_600_ of ~1.1 in 2 mL of sodium acetate buffer (20 mM, pH 4.6). Cultures were washed twice in sodium acetate buffer before being resuspended in 0.5 mL sodium acetate buffer with 0.25 mg/mL cytochrome C. Resuspended pellets were incubated while shaking at 37°C for 15 min, then centrifuged at 1,600 × *g* for 2 min. The supernatant was removed and aliquoted into 96-well plates, where the absorbance was measured at 410 nm using a Biotek plate reader. The percentage of cytochrome C bound for a given sample was calculated as the fraction of absorbance relative to 0.25 mg/mL cytochrome C in sodium acetate buffer alone.

### Membrane potential measurements

Membrane potential was assessed using DiSC3(5) (MedChem Express #HY-D0085-25mg) and a Biotek plate reader according to previously published methods (58). Cells were grown overnight to stationary phase, at which point they were diluted to an OD600 of 0.3. Cells were then incubated in 100 μL volumes in a black polystyrene 96-well plate with 5 μM DiSC3(5) for 3 min before being transferred to the plate reader. For cells treated with gramicidin, 1 μM gramicidin was added after the 3 min incubation of DiSC3(5), then read in the plate reader. Fluorescence was read in the plate reader at 610 nm excitation and 660 nm emission. Readings were conducted every 2.5 min for 1 h. Arbitrary fluorescence units were calculated by dividing the fluorescence measurement by the OD600 of the cells.

### Chemically defined media lacking excess potassium

To measure the effect of specific amounts of potassium in the growth media, we constructed chemically defined media lacking excess potassium as described previously (33). Defined amounts of potassium were added into the media by the addition of KCl. Survival assays were conducted at different KCl concentrations as described below.

### Survival assays

Overnight cultures were harvested, adjusted to a desired OD600, and incubated with an antibacterial compound of interest following the same protocol described above for MIC assays. Bacterial survival in various compounds was measured by plating and enumerating CFUs over the course of 24 h of static incubation at 37°C.

## SUPPLEMENTAL MATERIAL

Supplemental material msphere.00613-24-s0001.pdf Supplemental figures and tables.

ASM does not own the copyrights to Supplemental Material that may be linked to, or accessed through, an article. The authors have granted ASM a non-exclusive, world-wide license to publish the Supplemental Material files. Please contact the corresponding author directly for reuse.
