# Supplementary material for: Evolution of polyamine resistance in Staphylococcus aureus through modulation of potassium transport
Source: mSphere. 2025 Aug 18;10(9):e00613-24. doi: 10.1128/msphere.00613-24 (PMC12482181; doi:10.1128/msphere.00613-24)
Supplement: Supplemental material — Supplemental figures and tables. [file msphere.00613-24-s0001.pdf]

0.1 mM KCl Growth Curves

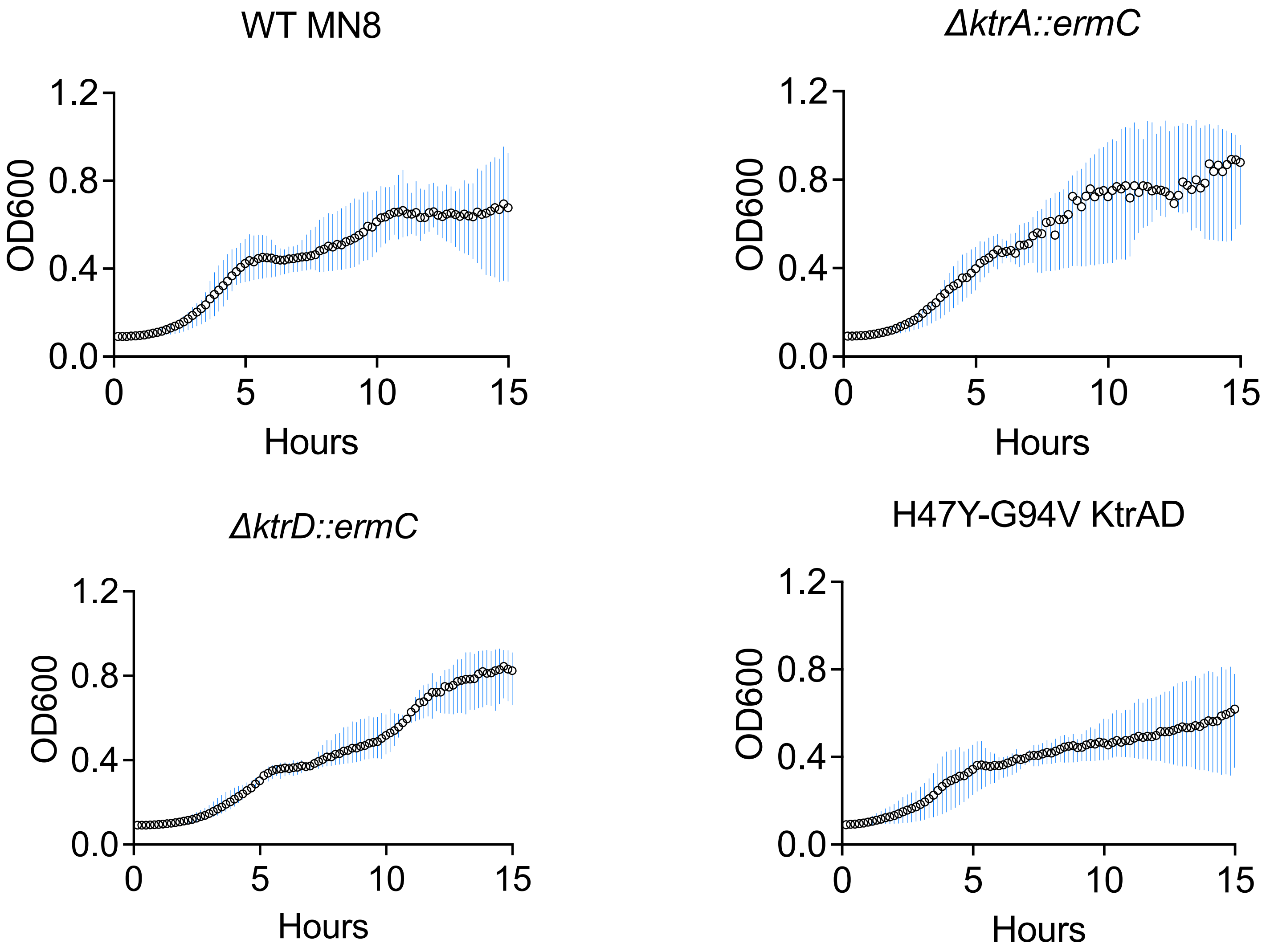

Supplementary Figure 1: Growth curves in defined media with 0.1mM KCl added in. Dots and lines on graphs represent mean and SEM of three independent experiments.

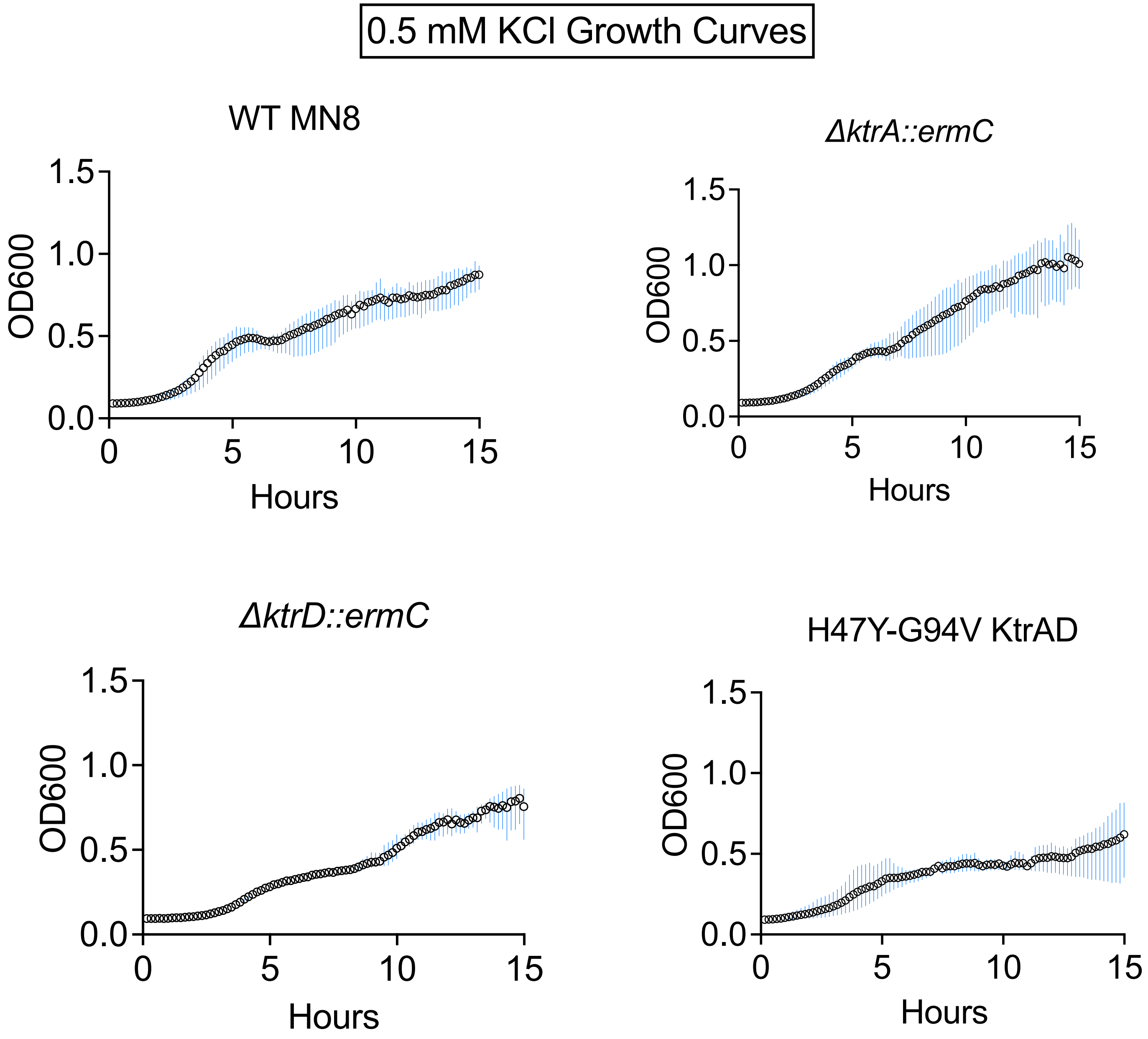

Supplemental figure 2: Growth curves in defined media with 0.5mM KCl added in. Dots and lines on graphs represent mean and SEM of three independent experiments.

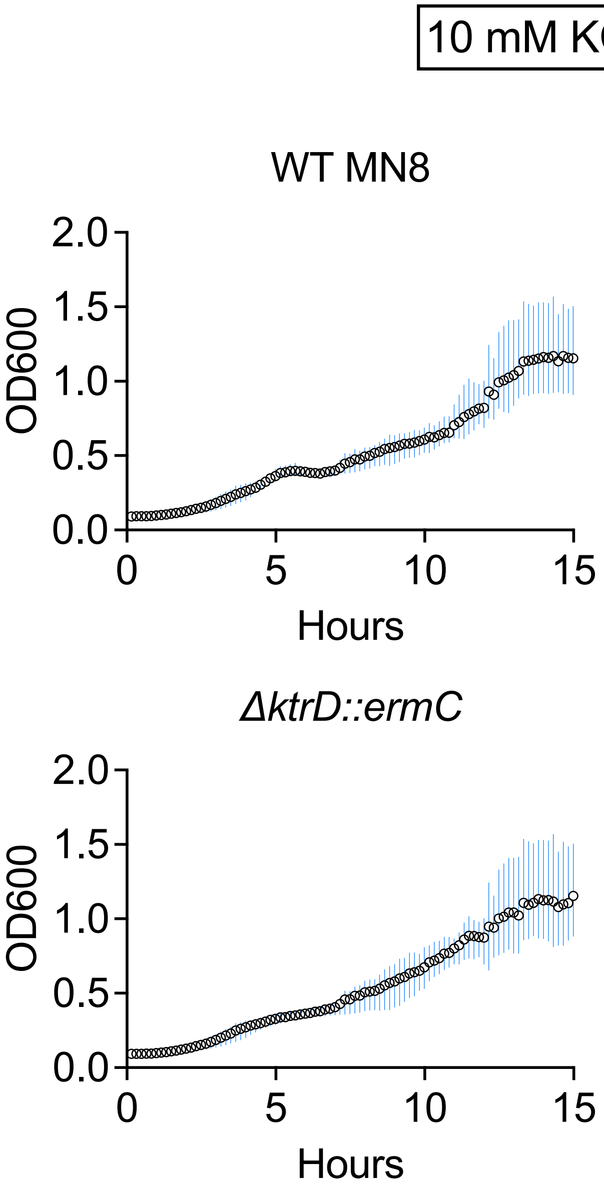

Supplemental figure 3: Growth curves in defined media with 10 mM KCl added in. Dots and lines on graphs represent mean and SEM of three independent experiments.

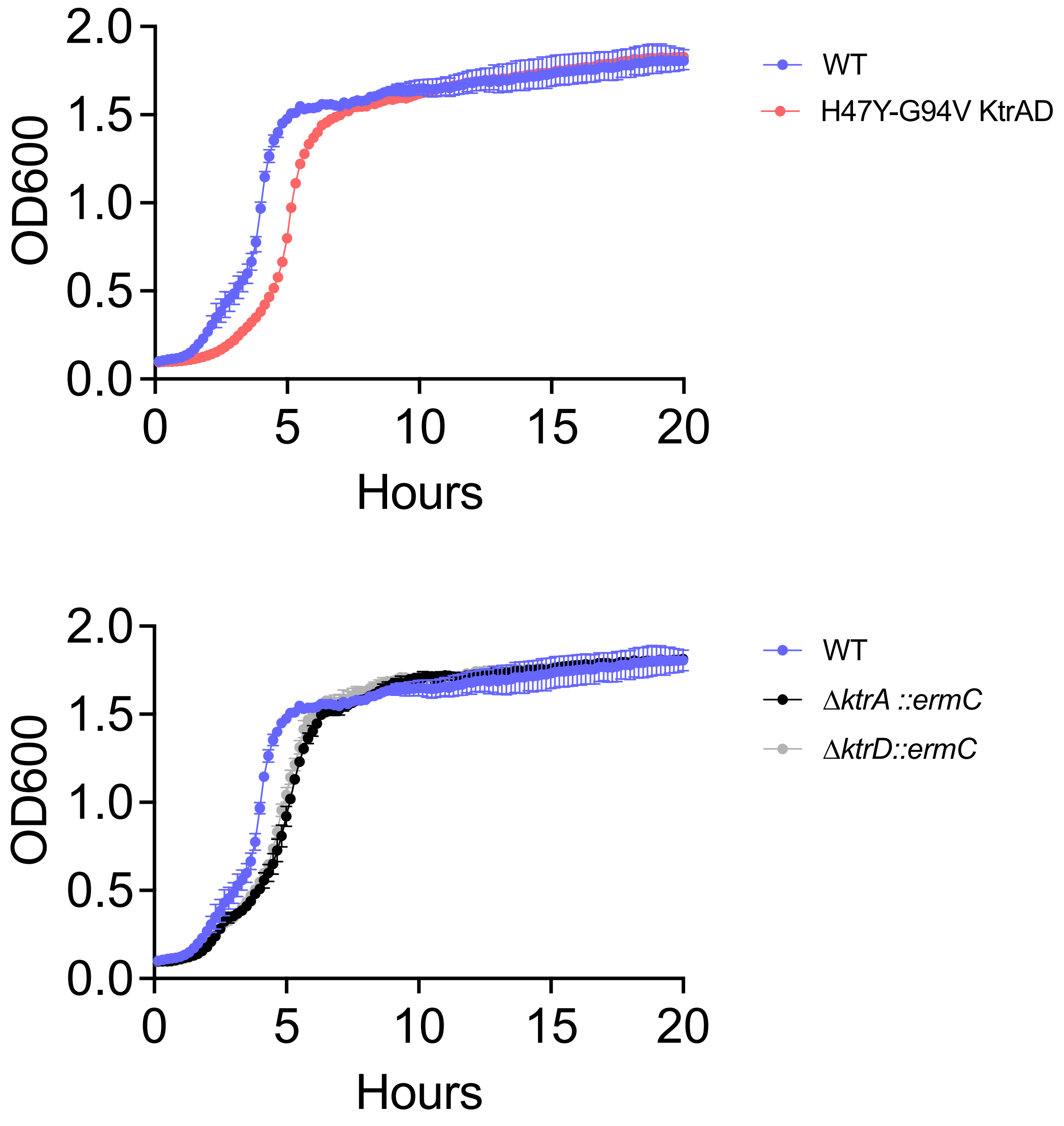

Supplemental figure 4: Growth curves of different Ktr mutants in TSB. Graphs represent mean and range of average OD measurements from 3 independent experiments.

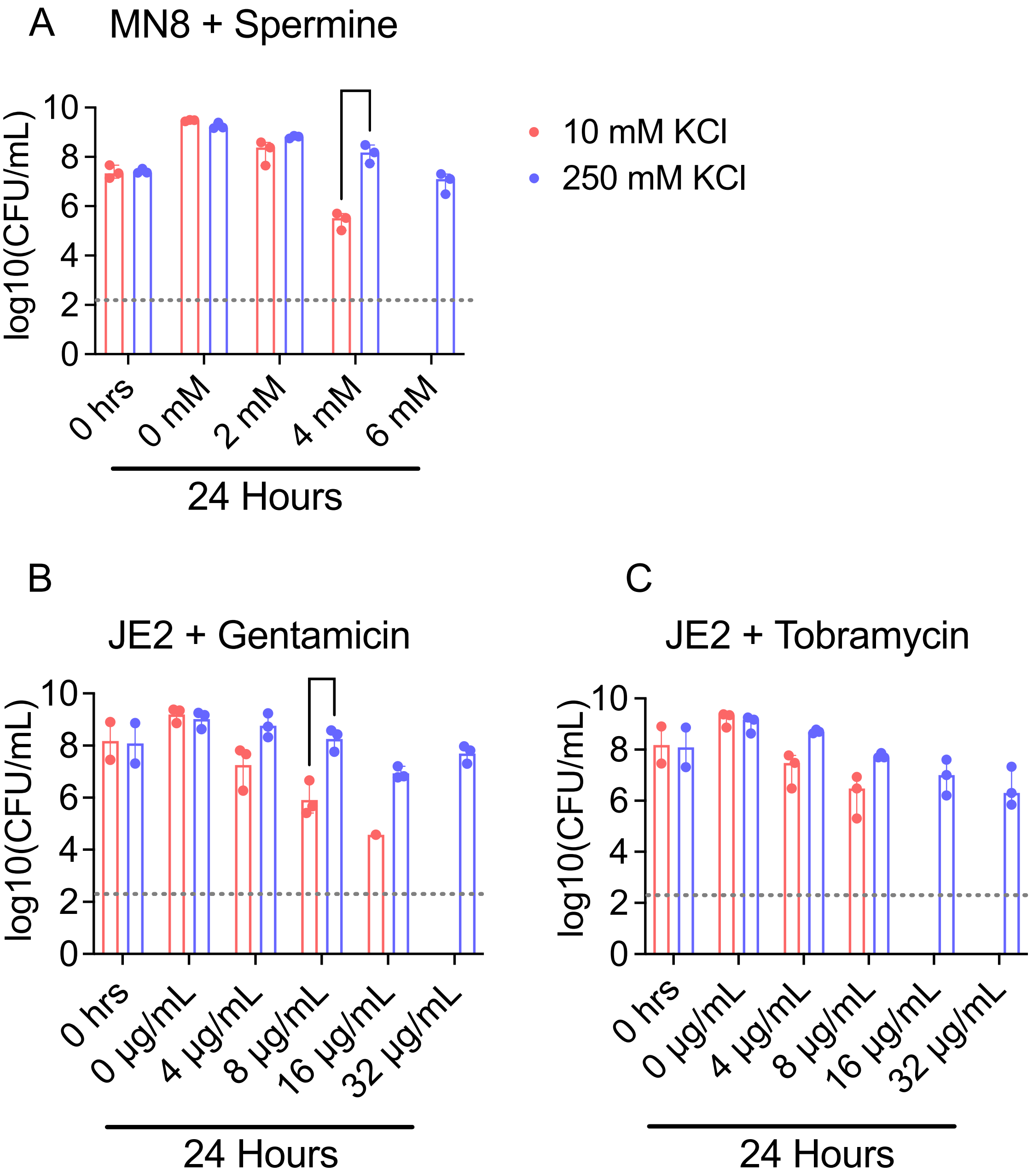

D

	10 mM KCl	250 mM KCl
Gentamicin	4-8	8-16
Tobramycin	0-2	4-8
Kanamycin	8-16	32-64

Supplemental figure 5: Effects of supraphysiological potassium concentrations on antimicrobial susceptibility

Enumeration of CFUs after incubation of S. aureus strain MN8 with spermine (A) or S. aureus strain JE2 with aminoglycosides in the presence of exogenous KCl (B,C). Gray dotted line denotes limit of detection. Asterisks denote significant difference in means between two treatments as detected by an unpaired t-test (P<0.05). No CFUs were detected for JE2 in 10mM KCl treated with aminoglycosides at concentrations above 8 micrograms/mL. Table (D) depicting MIC values in microgram/mL for different aminoglycoside antibiotics incubated for 24 hours with MN8 in different amounts of KCl.

Passage	Spermine exposure (mM)	Passage saved?
0	0	yes
1	2 mM	no
2	2 mM	no
3	2 mM	no
4	4 mM	yes
5	6 mM	no
6	6 mM	yes
7	6 mM	no
8	6 mM	yes
9	7 mM	no
10	7 mM	yes

Supplemental table 1: Spermine concentrations added at each passage for all spermine-exposed replicates.
Spermine concentrations that experimentally evolved populations were exposed to at each passage of the evolution experiment. Spermine was added to the final concentration directly in the growth media.

Replicate Line	*ktrA*	*ktrD*
1	H47Y	G94V
2	-	-
3	E76G	M391I
Control	-	-

Supplemental Table 2: Ktr complex Mutations that arose in *S. aureus* MN8 *ktrA* and *ktrD* during the pilot evolutionary experiment.

Strain	Source	Details
S. aureus MN8	BEI resources	https://www.beiresources.org/Catalog/bacteria/HM-162.aspx
S. aureus HFH-30364	BEI resources	https://beiresources.org/Catalog/bacteria/NR-10189.aspx
S. aureus RN4850	BEI resources	https://www.beiresources.org/Catalog/bacteria/NR-45955.aspx
S. aureus MN8 KtrA:H47Y	This study	Tyrosine substituted for histidine at the 47th position of the KtrA protein in the MN8 strain background
S. aureus MN8 KtrA:G94V	This study	Valine substituted for glycine at the 94thth position of the KtrA protein in the MN8 strain background
S. aureus MN8 KtrA:H47Y-G94V	This study	Tyrosine substituted for histidine at the 47th position of the KtrA protein and valine substituted for glycine at the 94thth position of the KtrA protein in the MN8 strain background
S. aureus MN8 *ktrA::ermC*	This study	*ktrA* gene replaced with erythromycin resistance gene *ermC*
S. aureus MN8 *ktrD::ermC*	This study	*ktrD* gene replaced with erythromycin resistance gene *ermC*

Supplemental Table 3:
List of *S. aureus* strains and their sources used in this study. Single amino acid mutations were introduced into the MN8 parent strain by allelic exchange as outlined in the methods.

Gene	Allele	Strain	Metadata	Sequence ID
ktrA	**A178V**	n/a	clinical or host-associated sample of *S. aureus*	HDB4168921.1
ktrA	A178T	SA-71	n/a	HDB2454818.1
ktrA	**E151V**	CFSA200	clinical or host-associated sample from *S. aureus*	WP_086896505.1
ktrA	E151K	n/a	clinical or host-associated sample from *S. aureus*	HDB5855606.1
ktrA	E76K	SMC9559	Generic sample Host: *Homo sapiens*	WP_217801415.1
ktrA		AF254	clinical or host-associated sample of *S. aureus*	MBV2625534.1
ktrA	**H47Y**	C51	Pig isolate	WP_031790491.1
ktrA		DAR3583	Generic sample Host: *Homo sapiens*	WP_031790491.1
ktrA		RIVM_M084907	Pathogen: clinical or host-associated sample from Staphylococcus aureus	HDE6238165.1
ktrA		RIVM_M085009	Pathogen: clinical or host-associated sample from Staphylococcus aureus	HDE6260146.1
ktrA		RIVM_M043274	Pathogen: clinical or host-associated sample from Staphylococcus aureus	HDF4313963.1
ktrD	**G94V**	H88163	Generic sample Host: *Homo sapiens*	WP_031807927.1
ktrD	G94D	M8158	Pathogen: clinical or host-associated sample from Staphylococcus aureus	HDA5546730.1
ktrD	**Q450K**	CM60	Pathogen: clinical or host-associated sample from Staphylococcus aureus	WP_111088627.1

Supplemental table 4: Ktr complex alleles in published sequencing data. List of mutations in publicly available sequences of *S. aureus* isolates in *ktrA* and *ktrD* genes. Queries were conducted using the identical protein group database on NCBI

Mutation	Frequency in population	Gene Annotation	Annotated Function
V348L (GTA→CTA)	7.50%	*clpB*	ATP-dependent chaperone protein ClpB
R51T (AGG→ACG)	8.20%	*lipA*	lipoyl synthase
L284I (TTA→ATA)	7.70%	*dnaB*	replicative DNA helicase
V384L (GTA→CTA)	6.10%	*nhaC*	Na+/H+ antiporter family protein
K56* (AAA→TAA)	10.4%	*traE*	Transfer complex protein
E76G (GAA→GGA)	100%	*ktrA*	potassium transport protein regulator
Q450K (CAA→AAA)	26.00%	*ktrD*	potassium transport protein
S135L (TCA→TTA)	61.90%	*ktrD*	potassium transport protein

Supplemental table 5: All present mutations and their frequencies in passage 10 of of the evolution experiment for population MN8-1.

Mutation (amino acid)	Frequency in population	Gene Annotation	Annotated Function
V261V (GTA→GTT)	11.00%	*rpoC*	DNA-directed RNA polymerase, beta' subunit
K283* (AAA→TAA)	5.30%	*dnaB*	replicative DNA helicase
R114S (AGA→AGT)	6.70%	*topB*	DNA topoisomerase
Y43C (TAC→TGC)	39.60%	*apt*	adenine phosphoribosyl transferase
S136P (TCT→CCT)	100%	hypothetical protein (Pan-genome locus tag: SAUPAN003400000)	predicted: XrtN system VIT domain protein
T366N (ACT→AAT)	10.80%	*pbp3*	penicillin-binding protein, transpeptidase domain protein
M1K (ATG→AAG) †	5.70%	*purQ*	phosphoribosylformylglycinamidine synthase I
N86K (AAT→AAA)		*purS*	phosphoribosylformylglycinamidine synthase, purS protein

Supplemental table 6: All present mutations and their frequencies in passage 10 of of the evolution experiment for population MN8-2

Mutation	Frequency in population	Gene Annotation	Function
S247C (AGC→TGC)	20.80%	hypothetical protein	DUF536 domain-containing protein
E76G (GAA→GGA)	84.00%	*ktrA*	potassium transport channel regulator
M391I (ATG→ATA)	16.60%	*ktrD*	potassium transport channel
T277N (ACT→AAT)	59.00%	*ktrD*	potassium transport channel

Supplemental table 7: All present mutations and their frequencies in passage 10 of of the evolution experiment for population MN8-3.

Mutation	Frequency in population	Gene Annotation	Function
N28K (AAT→AAA)	6.1%	*ccpA*	catabolite control protein A
L284I (TTA→ATA)	9.3%	*dnaB*	replicative DNA helicase
T278S (ACT→TCT)	8.7%	*dnaB*	replicative DNA helicase
A178V (GCA→GTA)	100.00%	*ktrA*	potassium channel regulator
G94C (GGT→TGT)	100.00%	*ktrD*	potassium transport protein

Supplemental table 8: All present mutations and their frequencies in passage 10 of of the evolution experiment for population MN8-4.

Mutation	Frequency in population	Gene Annotation	Function
G4E (CGA→GAA)	37.1%	*pgl*	6-phosphogluconolactonase
H306Y (CAT→TAT)	60.2%	*pgl*	6-phosphogluconolactonase
P95L (CCG→CTG)	56.8%	*atpA*	atp synthase alpha subunit
M656L (ATG→TTG)	8.1%	*purK*	N5-carboxyaminoimidazole ribonucleotide synthase

Supplemental table 9: All present mutations and their frequencies in passage 10 of of the evolution experiment for population HFH-1.

Mutation	Frequency in population	Gene Annotation	Function
D222Y (GAC→TAC)	7.1%	*mprF*	Phosphatidylglycerol lysyltransferase
G287V (GGT→GTT)	75.3%	*pgl*	6-phosphogluconolactonase
G323D (GGT→GAT	20.9%	*pgl*	6-phosphogluconolactonase

Supplemental table 10: All present mutations and their frequencies in passage 10 of of the evolution experiment for population HFH-3.

Mutation	Frequency in population	Gene Annotation	Function
G4E (CGA→GAA)	5.6%	*pgl*	6-phosphogluconolactonase
G120S (GGT→AGT)	75.3%	*pgl*	6-phosphogluconolactonase
R318S (AGA→AGC)	91.1%	*pgl*	6-phosphogluconolactonase
G69R (GGC→CGC)	100%	*lipR*	lysR-type transcriptional regulator

Supplemental table 11: All present mutations and their frequencies in passage 10 of of the evolution experiment for population HFH-4.

Mutation	Frequency in population	Gene Annotation	Function
L142L (TTA→CTA)	19.7 %	*arcC*	carbamate kinase
Q99* (CAG→TAG)	5.4 %	*mhqA*	Putative ring-cleaving dioxygenase MhqA
C217F (TGT→TTT)	5.1%	*mprF*	phosphatidylglycerol lysyltransferase

Supplemental table 12: All present mutations and their frequencies in passage 10 of the evolution experiment for populations HFH-5 and HFH-6.

Mutations	Frequency in population	Gene Annotation	Function
A245V (GCA→GTA)	82.4 %	*pgl*	6-phosphogluconolactonase
T10P (ACT→CCT)	13.7%	*pgl*	6-phosphogluconolactonase
Q99* (CAG→TAG)	5.4 %	*mhqA*	Putative ring-cleaving dioxygenase MhqA
F114L (TTT→TTA)	100 %	hypothetical protein	predicted: XrtN system VIT domain protein
T150I (ACT→ATT)	100 %	*bsbh2*	bacillithiol biosynthesis deacetylase BshB2
S147T (AGT→ACT)	13.8%	hypothetical, Pan-locus tag: SAUPAN001647000	phi ETA orf 22-like protein
Q312H (CAG→CAC)	12.6%	*fakA*	fatty acid kinase
S111T (AGT→ACT)	12.2%	hypothetical protein	DUF669 domain-containing protein
A31P (GCA→CCA)	6.8%	*ccpA*	catabolite control protein A
A161P (GCC→CCC)	6.3%	dnaD containing protein	DNA replication initiation protein
R577P (CGC→CCC)	6.2%	*priA*	primosomal protein N helicase

Supplemental table 13: All present mutations and their frequencies in passage 10 of of the evolution experiment for population RN-1.

Mutation	Frequency in population	Gene Annotation	Function
P234L(CCT→CTT)	45.7%	*pgl*	6-phosphogluconolactonase
S41F(TCT→TTT)	10.4%	*pgl*	6-phosphogluconolactonase
G261E (GGG→GAG)	100%	*pgl*	6-phosphogluconolactonase
H138Y (CAT→TAT)	9.3%	*pgl*	6-phosphogluconolactonase
F268V (TTT→GTT)	8.1%	*pgl*	6-phosphogluconolactonase
D216H (GAT→CAT)	6.3%	*cobI*	magnesium transport protein
Δ69 bp	8.3%	*atpG*	atp synthase subunit gamma
R84T (AGA→ACA)	10.7%	*nudF*	nudix hydrolase
D100H (GAT→CAT)	9.6%	hypothetical (*S. aureus* Pan-locus tag: SAUPAN001824000)	phiSLT ORF116b-like protein
A86P (GCA→CCA)	9.5%	*gluD*	glutamate dehydrogenase
A159P (GCG→CCG)	9.3%	dnaD containing protein	DNA replication initiation protein
E68Q (GAG→CAG)	6.1%	hypothetical protein (Pan-locus tag: SAUPAN004460000)	pyridine nucleotide-disulfide oxidoreductase

Supplemental table 14: All present mutations and their frequencies in passage 10 of of the evolution experiment for population RN-2.

Mutations	Frequency in population	Gene Annotation	Function
A245V (GCA→GTA)	31.5%	*pgl*	6-phosphglucolactonase
N143D (AAT→GAT)	52.9%	hypothetical protein (Pan-genome locus tag: SAUPAN003400000)	predicted: XrtN system VIT domain protein
H105Y (CAT→TAT)	39.8%	hypothetical protein (Pan-locus tag: SAUPAN006167000)	hypothetical protein
S136P (TCT→CCT)	39.7%	hypothetical protein	predicted: XrtN system VIT domain protein
S163C (TCT→TGT)	9.6%	*bsaA*	glutathione peroxidase
L261* (TTA→TGA)	6.7%	*atpG*	atp synthase subunit gamma
G207A (GGC→GCC)	5.5%	*capE*	capsular polysaccharide synthesis gene 5E
V173L (GTC→CTC	5.3%	*epiE*	lantibiotic ABC transporter

Supplemental table 15: All present mutations and their frequencies in passage 10 of of the evolution experiment for population RN-3.

Mutations	Frequency in population	Gene Annotation	Function
S136P (TCT→CCT)	39.7%	hypothetical protein (Pan-genome locus tag: SAUPAN003400000)	predicted: XrtN system VIT domain protein
R116C (CGT→TGT)	28.5%	*sucC*	succinyl-CoA synthetase subunit beta
Δ1 bp	100%	*recJ*	single-stranded DNA exonuclease
Δ6 bp	74.5%	*rpoA*	DNA-directed RNA polymerase subunit alpha
Q170*	14.8%	*atpH*	atp synthase subunit delta
L213* (TTA→TAA)	14.5%	*corA*	magnesium transporter
V310L (GTA→CTA)	11.8%	hypothetical protein (Pan-genome locus: SAUPAN001539000)	phage major capsid protein
L242V (CTT→GTT)	10.10%	hypothetical protein (Pan-genome locus: SAUPAN001168000)	unknown function
S33T (AGC→ACC)	100%	hypothetical protein (locus tag: SAOUHSC_01579)	unknown function
W650S (TGG→TCG)	9.4%	hypothetical protein (Pan-genome tag: SAUPAN002859000)	unknown function
S147T (AGT→ACT)	8.7%	hypothetical (*S. aureus* Pan-locus tag: SAUPAN001824000)	phiSLT ORF116b-like protein
S163C (TCT→TGT)	7.9%	*bsaA*	glutathione peroxidase
A223P (GCT→CCT)	7.7%	*mprF*	phosphatidylglycerol lysyltransferase
E1220D (GAG→GAC)	7.3%	*essC*	type VII secretion protein EssC
Q68* (CAA→TAA)	7.2%	*htrA1*	serine protease
Y43C (TAC→TGC)	5.9%	*apt*	adenine phosphoribosyltransferase

Supplemental table 16: All present mutations and their frequencies in passage 10 of of the evolution experiment for population RN-4.
